# Supplementary material for: Soil fungi remain active and invest in storage compounds during drought independent of future climate conditions
Source: Nat Commun. 2024 Nov 29;15:10410. doi: 10.1038/s41467-024-54537-y (PMC11607446; doi:10.1038/s41467-024-54537-y)
Supplement: Supplementary file 1 — Supplementary Information [file 41467_2024_54537_MOESM1_ESM.pdf]

Supplementary Information File:

Soil fungi remain active and invest in storage compounds during drought independent of future climate conditions.

Author list:

Alberto Canarini[1,2*§], Lucia Fuchslueger[1,3*§], Jörg Schnecker[1], Dennis Metze[1,4], Daniel B. Nelson[5], Ansgar Kahmen[5], Margarete Watzka[1], Erich M. Pötsch[6], Andreas Schaumberger[6], Michael Bahn[7], Andreas Richter[1,8*]

Affiliations:

[1] Centre for Microbiology and Environmental Systems Science, University of Vienna, Vienna, Austria

[2] Department of Biological, Geological and Environmental Sciences, University of Bologna, Bologna, Italy

[3] Environment and Climate Hub, University of Vienna, Vienna, Austria

[4] Doctoral School in Microbiology and Environmental Science, University of Vienna, Vienna, Austria

[5] University of Basel, Department of Environmental Sciences – Botany, Basel, Switzerland

[6] Agricultural Research and Education Centre Raumberg-Gumpenstein, Irdning, Austria

[7] Department of Ecology, Universität Innsbruck, Innsbruck, Austria

[8] International Institute for Applied Systems Analysis (IIASA), Laxenburg, Austria

[§] These authors contributed equally to this work

Corresponding authors: * Alberto Canarini, Lucia Fuchslueger, Andreas Richter

Email: alberto.canarini@hotmail.it; lucia.fuchslueger@univie.ac.at; andreas.richter@univie.ac.at

This PDF file includes:

Supplementary Figures 1 to 14

Supplementary Tables 1 to 6

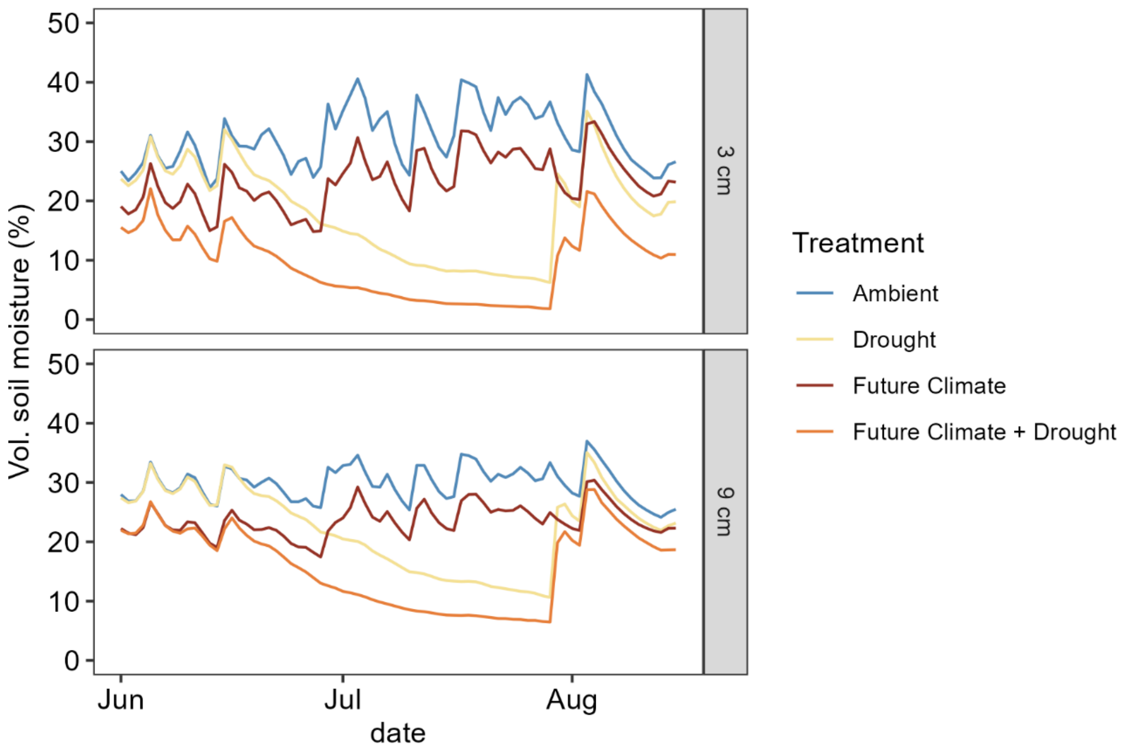

Supplementary Figure 1. Soil water content over the course of the experiment. Daily averages of volumetric soil moisture (%) at a) 3 and b) 9 cm of soil over the course of the experiment (June-August 2020). The line colours indicate the different treatments.

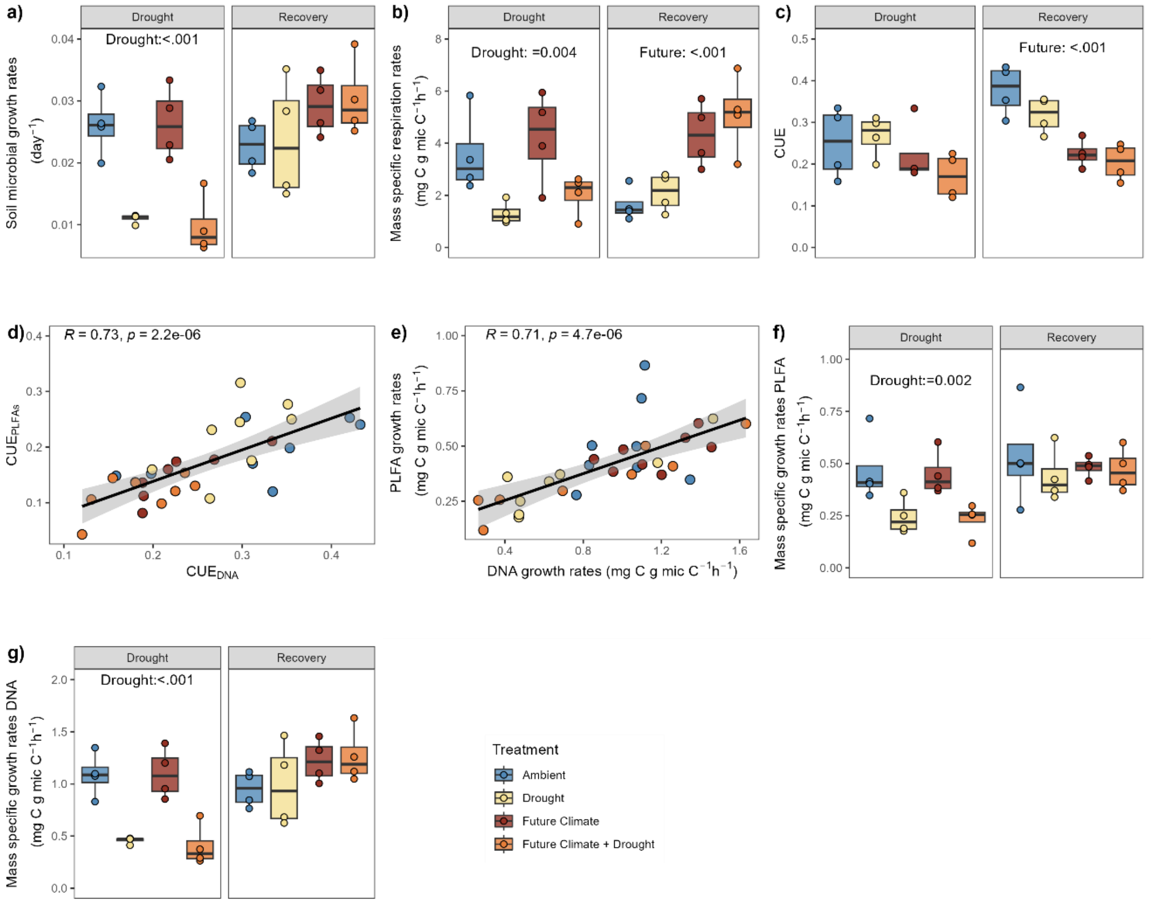

Supplementary Figure 2. Soil microbial community-level growth rates, respiration and CUE measured via^{18}O incorporation into DNA and correlation with ^{2}H incorporation into PLFA. a) Mass specific growth rates obtained via ^{18}O incorporation into DNA, b) respiration rates, as well as c) microbial CUE measured at Drought and Recovery. The correlations panels compare d) PLFA and DNA-based microbial community carbon use efficiency (CUE) and e) mass-specific growth rates, obtained by the two methods. Pearson's correlation coefficient R (and p-values) are reported on the graph. The last two panels represent mass specific growth rates (mg C g mic C^{-1} h^{-1}) obtained by each method, f) via ^{2}H incorporation into PLFA and g) via ^{18}O incorporation into DNA. In panel a, b, c, f and g) significant differences ($p<0.05$) between treatments derived from linear mixed models are reported in the figure (the full report is provided in Supplementary Table 2). Box centre line represents median, the box indicates the upper and lower quartiles, whiskers the 1.5x interquartile range, and separated points represent potential outliers. The sample size 'n' represents biologically independent samples (n=4 replicates). Colour indicates treatment.

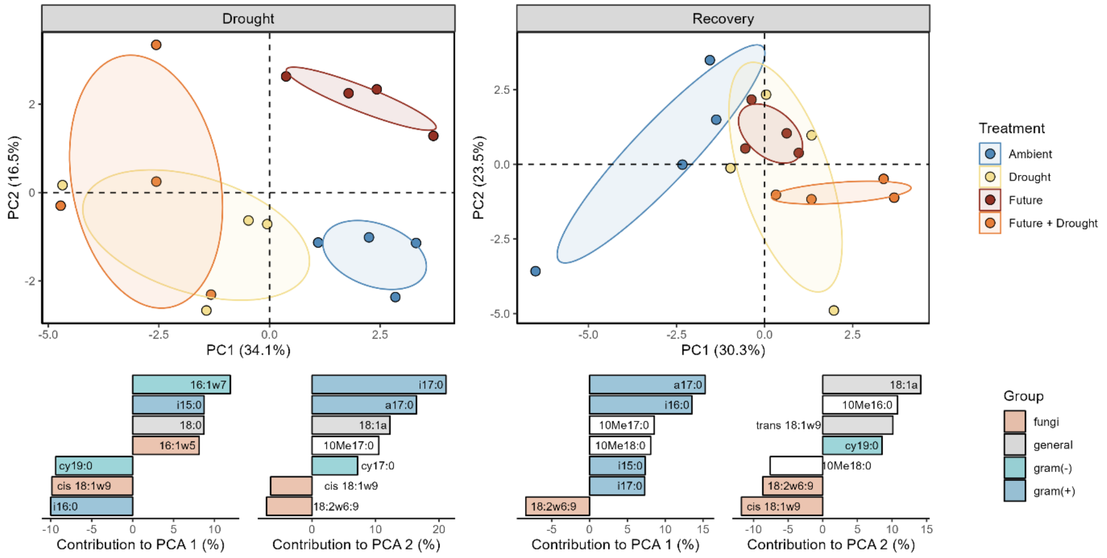

Supplementary Figure 3. Microbial community composition based on the relative abundance of individual PLFA biomarkers displayed as PCA during the Drought and Recovery period. Colour indicates treatment. Permanova results are reported in Supplementary Table 3. The sample size ‘n’ represents biologically independent samples (n=4 replicates). Ellipses represent the 95% confidence intervals. The bottom graphs represent the relative contribution (in percentage) of the top seven variables to the principal components (absolute values represent the relative contribution while the positive or negative sign represents the direction along PCA axes) colored according to microbial group (gram positive: blue; gram negative: light blue; general marker: grey; fungi: orange; actinobacteria: white; note that the arbuscular mycorrhizae fungal biomarker 16:1ω5 is included into the fungal group in the multivariate analysis but not in other graphs displaying fungi).

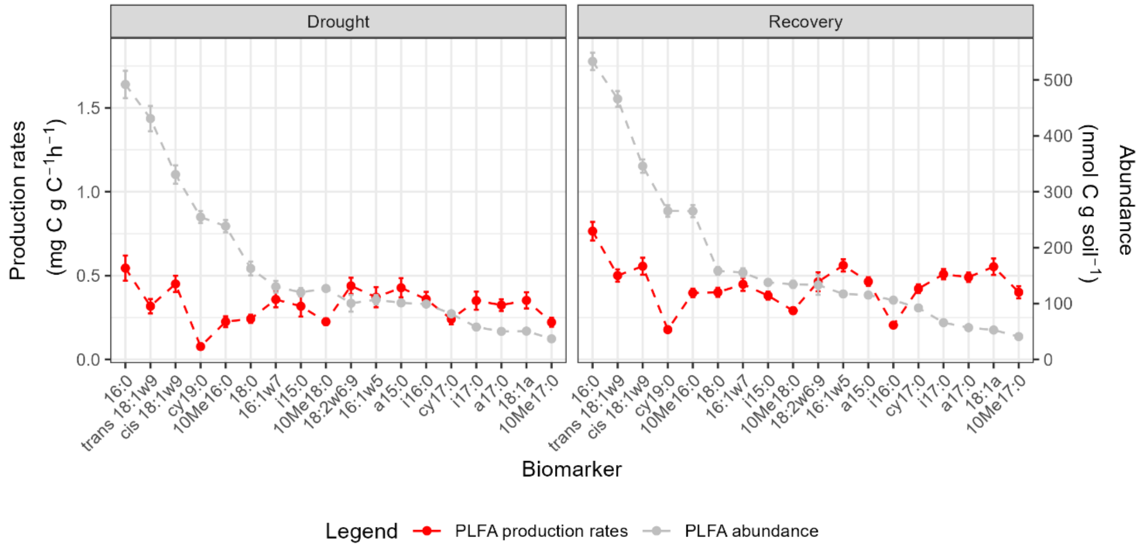

Supplementary Figure 4. Individual PLFA FAME biomarker contents (in nmol C g^{-1} soil, grey symbols), and respective production rates (in mg C g^{-1} PLFA C h^{-1}, red symbols) in the Drought and Recovery period. The right y-axis represents the absolute abundances, the left y-axis the production rates (or mass-specific growth rates). Fatty acids on the x-axis are ordered by ascending absolute abundance. Points represent averages across all treatments, error bars represent standard error. The sample size 'n' represents biologically independent samples (n=4 replicates).

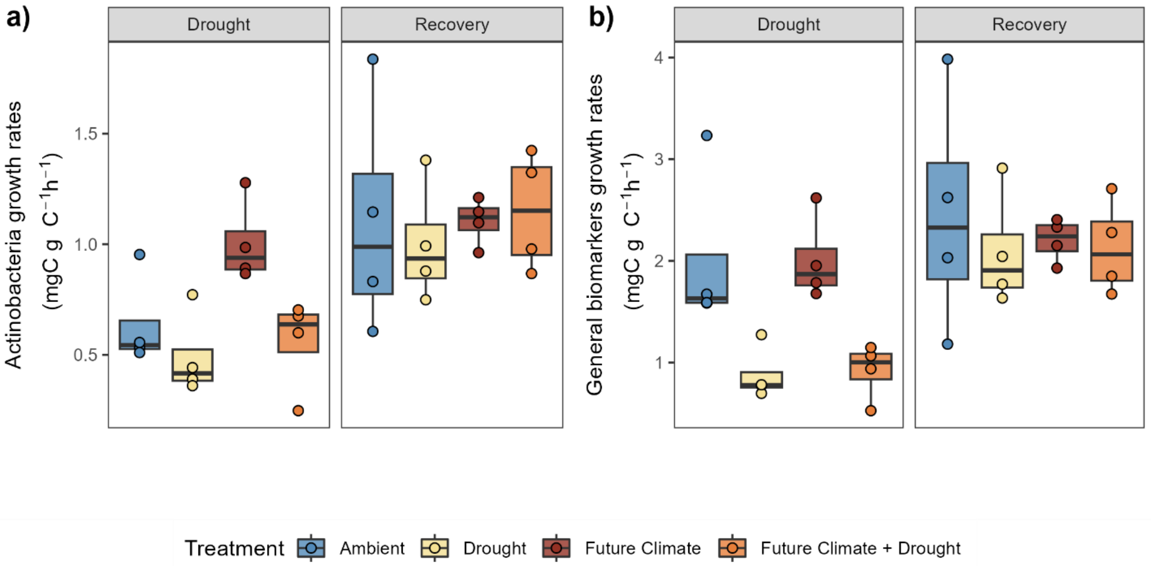

Supplementary Figure 5. Mass-specific growth rates of a) actinobacteria and b) general biomarkers, separately for peak drought (‘Drought) and Recovery. Statistical results are reported in the Supplementary Table 4. Box centre line represents median, box limits the upper and lower quartiles, whiskers the 1.5x interquartile range, while separated points represent potential outliers. The sample size ‘n’ represents biologically independent samples (n=4 replicates). For all the graphs colour indicates treatment.

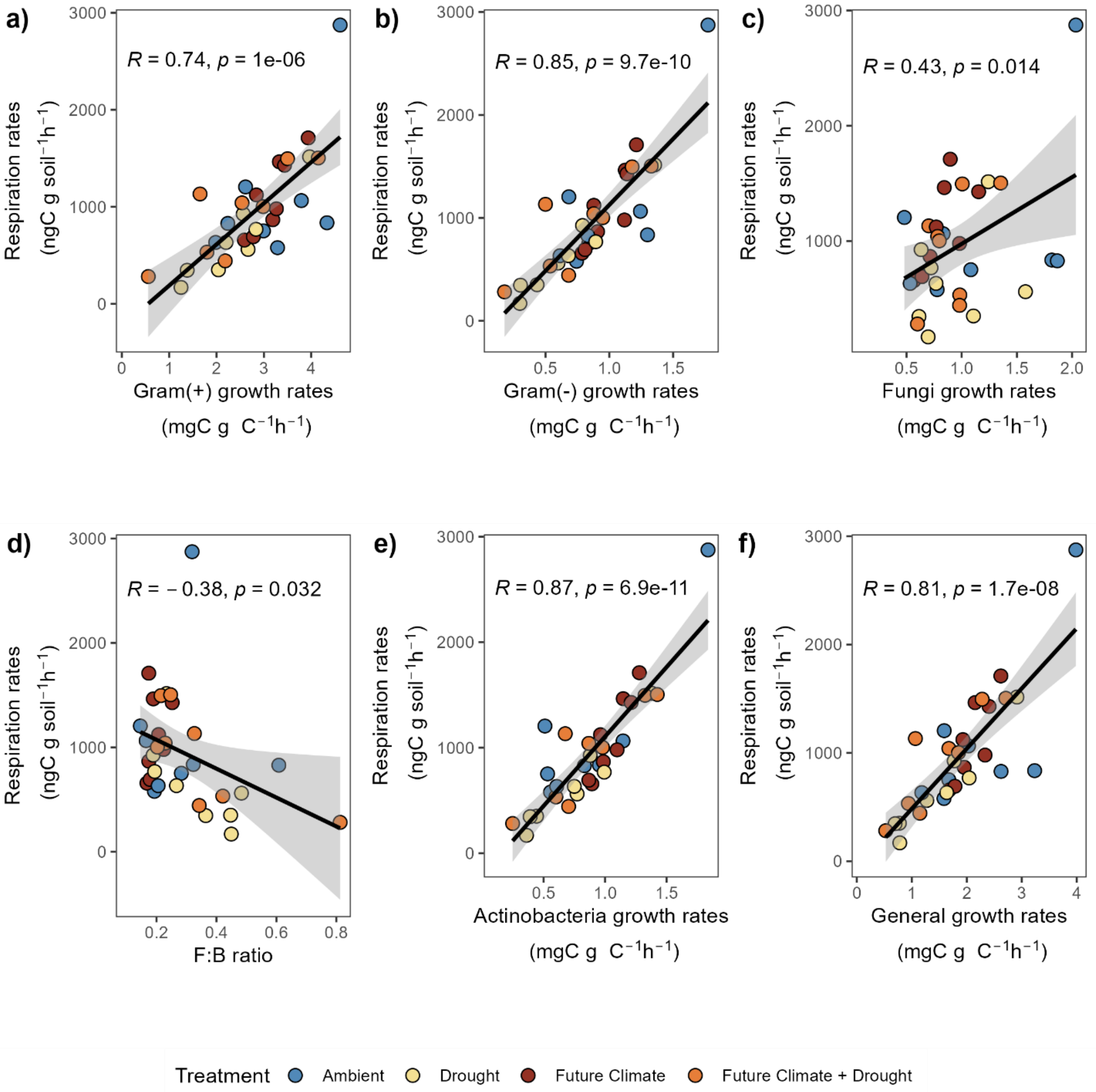

Supplementary Figure 6. Correlations between mass specific growth rates and total respiration rates of a) gram positive, b) gram negative, c) fungi, e) actinobacteria and f) general biomarkers. Correlation with fungi to bacteria ratio is shown in panel d). Correlation were taken across all the time points mesured (Drought and Recovery). Pearson correlation coefficient (R) and p-value are reported on each graph. For all the graphs color indicates treatment.

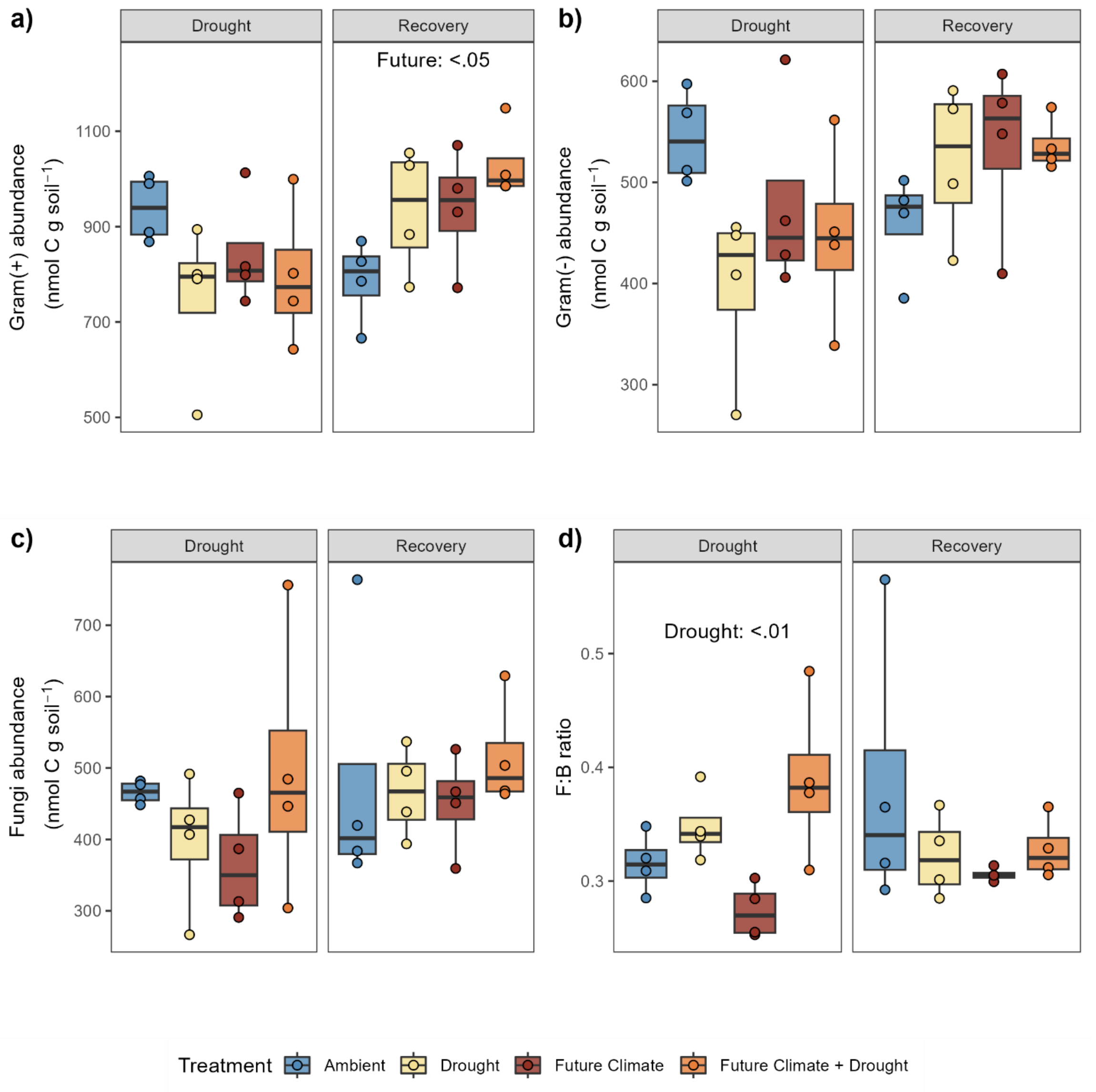

Supplementary Figure 7. PLFA-based biomass of microbial groups for a) Gram positive, b) Gram negative, c) fungi, and d) fungal to bacterial marker ratios, split in time points measured (Drought and Recovery). Statistical results are reported only for significant p-values. Box centre line represents median, box limits the upper and lower quartiles, whiskers the 1.5x interquartile range, while separated points represent potential outliers. The sample size 'n' represents biologically independent samples (n=4 replicates). For all the graphs colour indicates treatment.

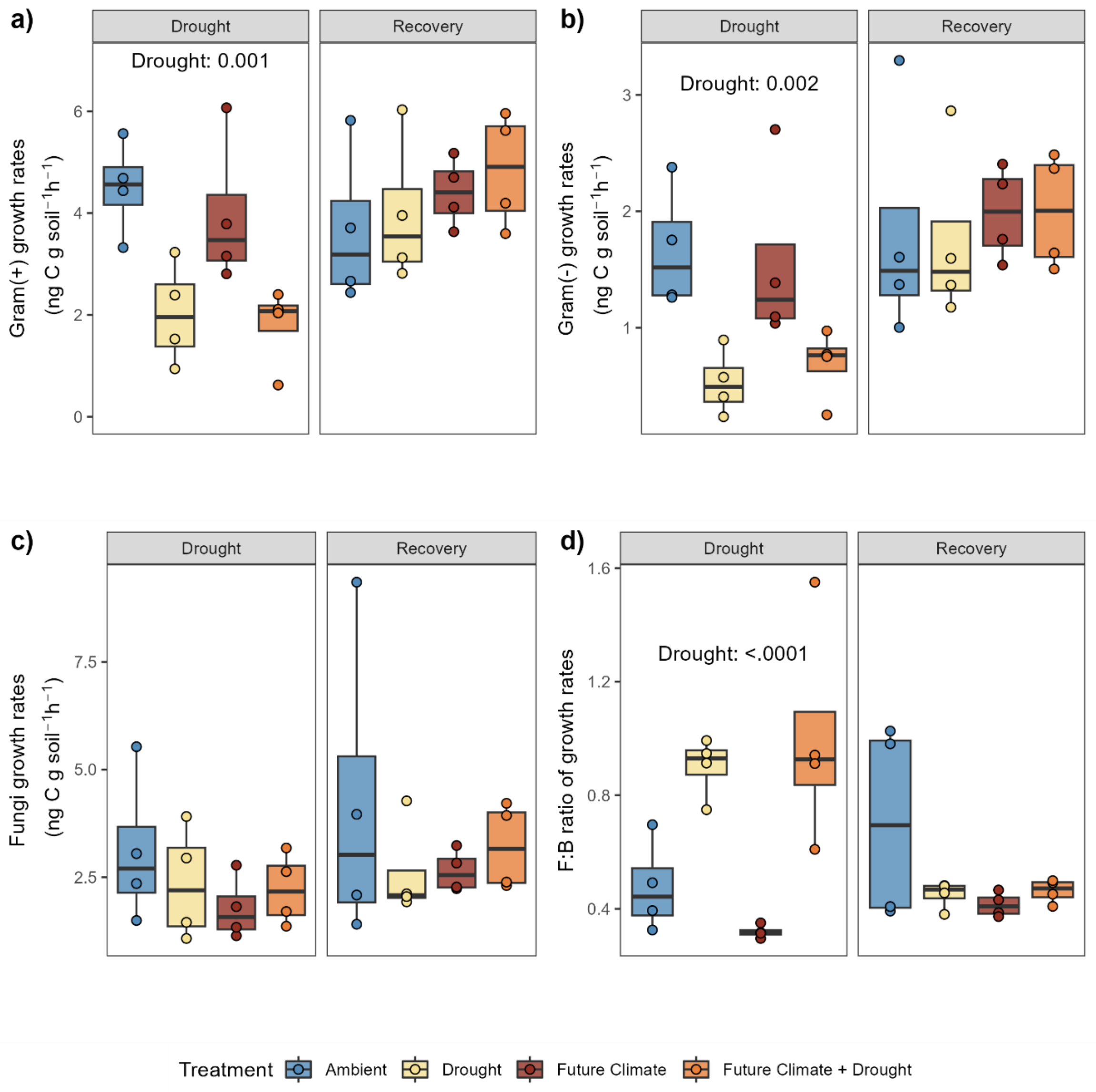

Supplementary Figure 8. Absolute growth rates of different microbial groups. a) Gram-positive and b) Gram-negative bacterial markers and c) fungal markers, as well as d) the ratio of fungal to bacterial growth rates at peak drought and recovery (Drought and Recovery). Statistical results are reported for significant p-values. Box centre line represents median, box limits the upper and lower quartiles, whiskers the 1.5x interquartile range, while separated points represent potential outliers (n=4 replicates per treatment). Colour indicates treatment.

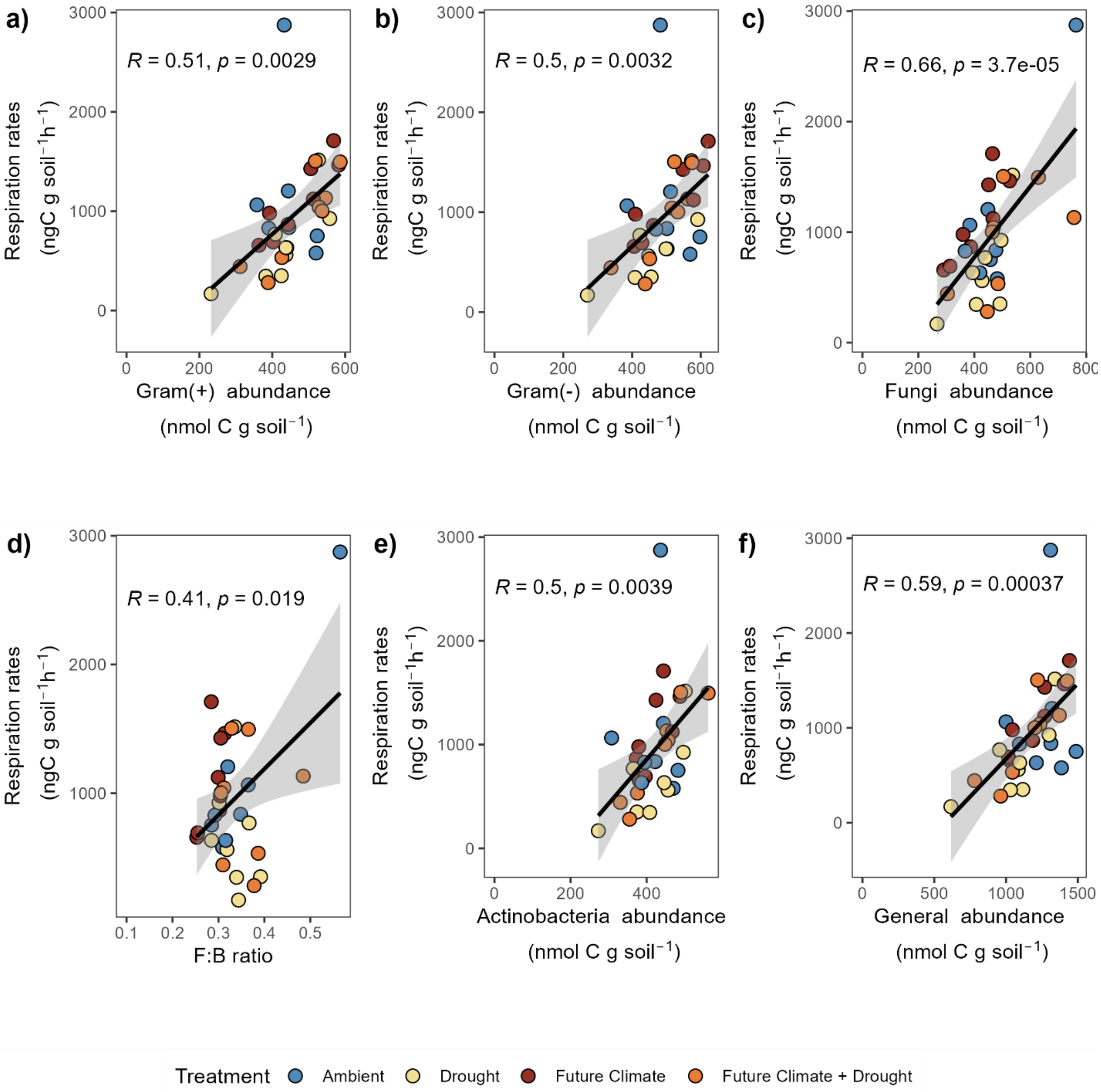

Supplementary Figure 9. Correlations between PLFA based microbial biomass separated by groups and total respiration rates of a) Gram positive, b) Gram negative, c) fungi, e) actinobacteria, and f) general biomarkers, d) shows the correlation of fungi to bacteria ratio with total respiration rates. Correlation were taken across all the time points mesured (Drought and Recovery). Pearson correlation coefficient (R) and p-value are reported on each graph. For all the graphs color indicates treatment.

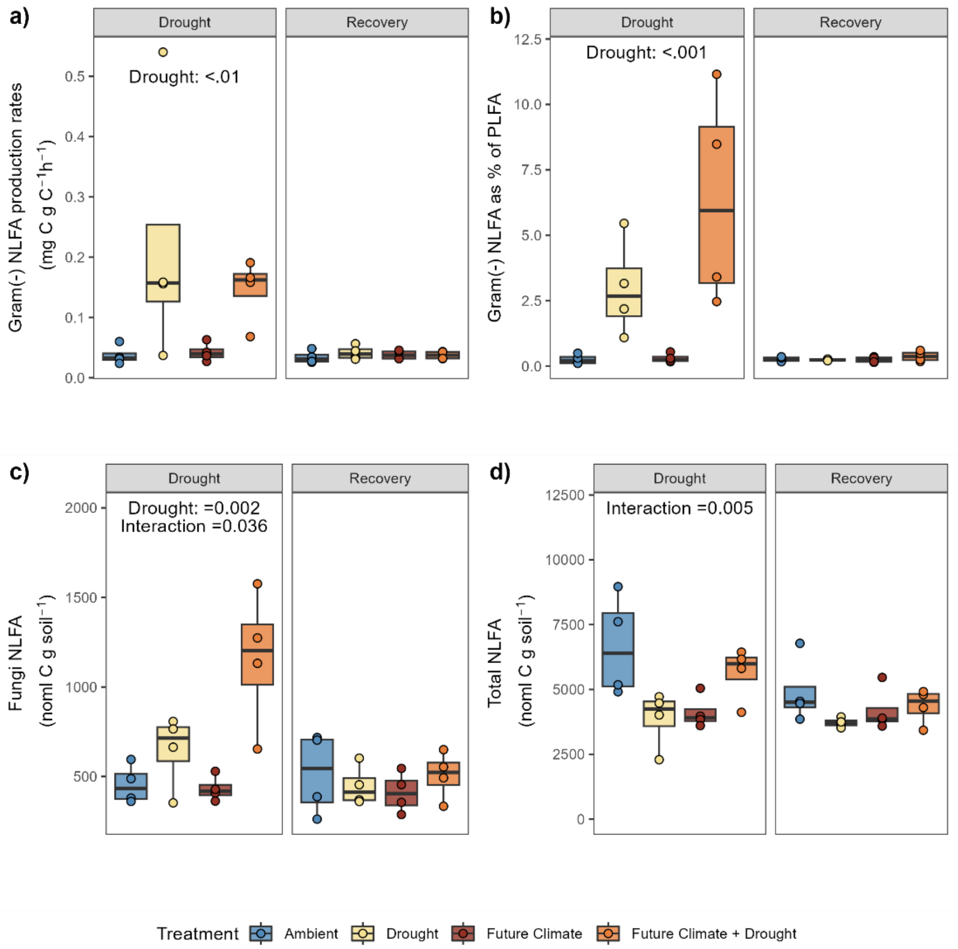

Supplementary Figure 10. Effects of drought and future climate on Gram negative NLFA production as well as total and fungal biomarker amounts. a) Production of gram negative specific NLFA biomarker; b) investment in NLFA relative to the same Gram-negative PLFA specific biomarkers (shown as percent); c) fungal specific biomarkers (as total amount) and d) total NLFA (sum of all biomarkers), divided by the time points measured (Drought and Recovery). Statistical results are reported for significant p-values (for a full report see Supplementary Table 5). Box center line represents median, box limits the upper and lower quartiles, whiskers the 1.5x interquartile range, while separated points represent potential outliers. The sample size ‘n’ represents biologically independent samples (n=4 replicates). Color indicates treatment.

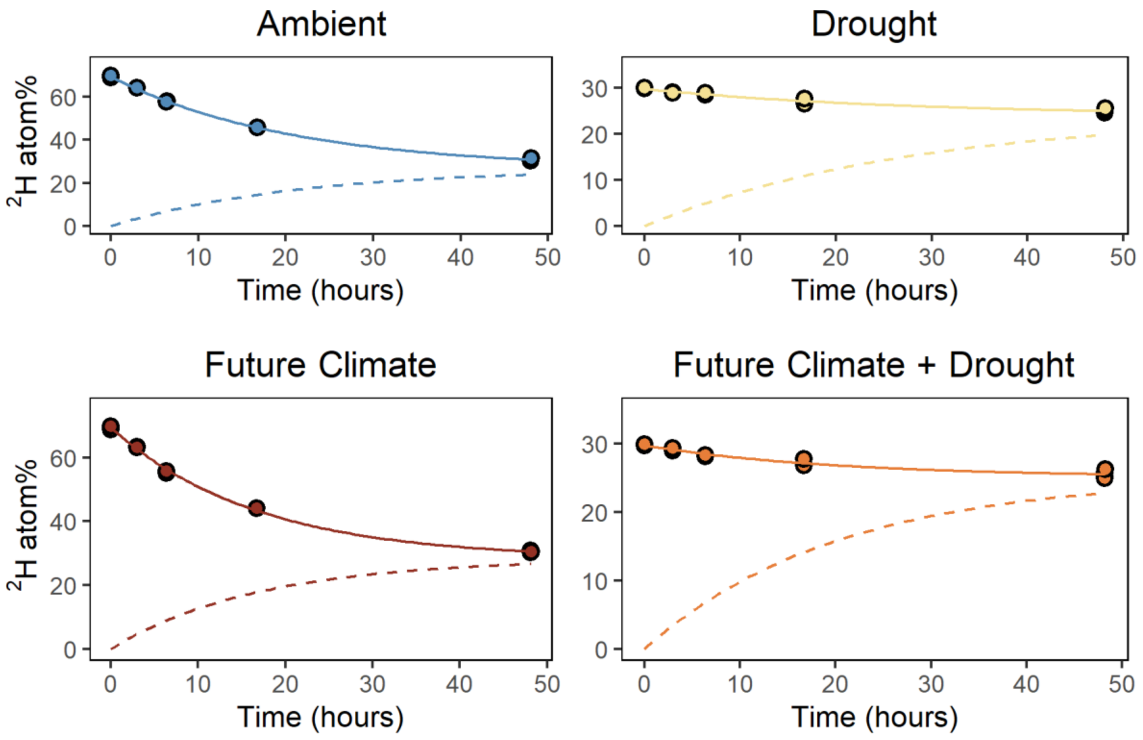

Supplementary Figure 11. Temporal dynamic of ^{2}H equilibration with soil water. Points represent measured ^{2}H atom% values in the ^{2}H labelled source water reaching isotopic equilibration rates of the external ^{2}H labelled source water with soil water of the four treatments. Continuous lines represent the best fit model. Dashed lines represent the model prediction of ^{2}H kinetics in the soil water, generated from the data points of the labelled source water, as described in Canarini et al. (2020) and the in Supplementary Methods.

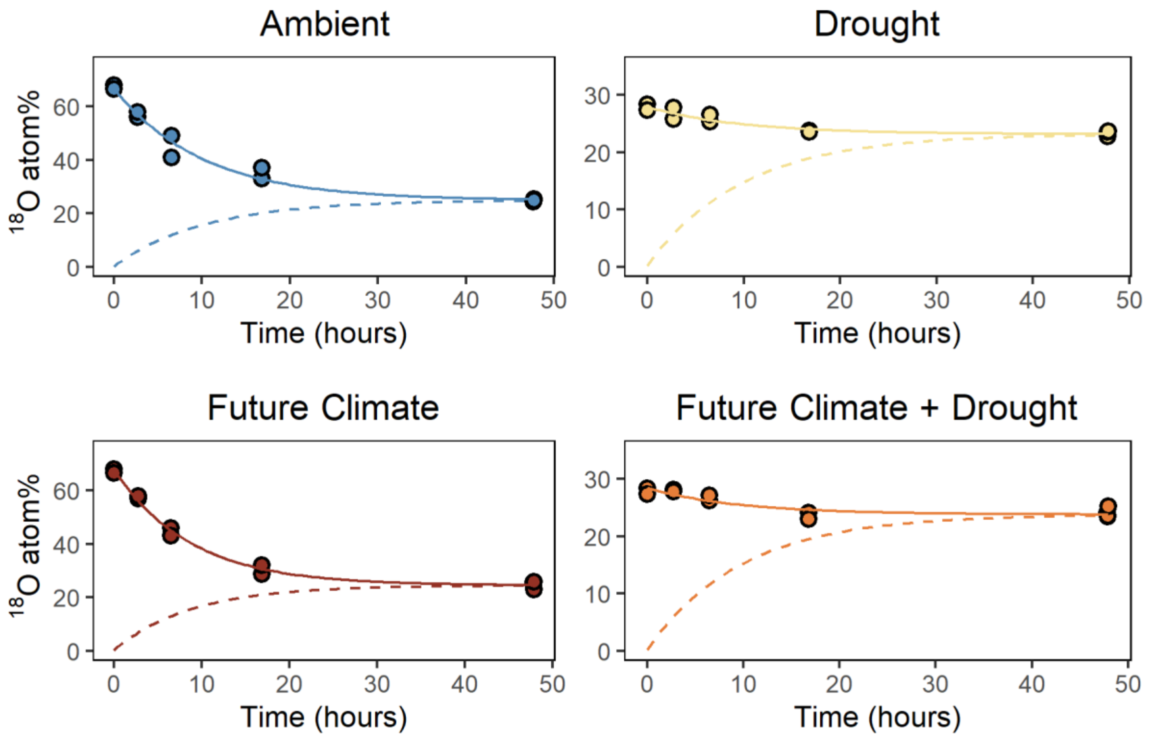

Supplementary Figure 12. Temporal dynamics of ^{18}O equilibration with soil water. Points represent measured ^{18}O atom% values in the ^{18}O labelled source water reaching isotopic equilibration rates of the external ^{18}O labelled source water with soil water of the four treatments. Continuous lines represent the best fit model, dashed lines represent the model prediction of ^{18}O kinetics in the soil water, generated from the data points of the labelled source water, as described in Canarini et al. (2020) and in the Supplementary Methods.

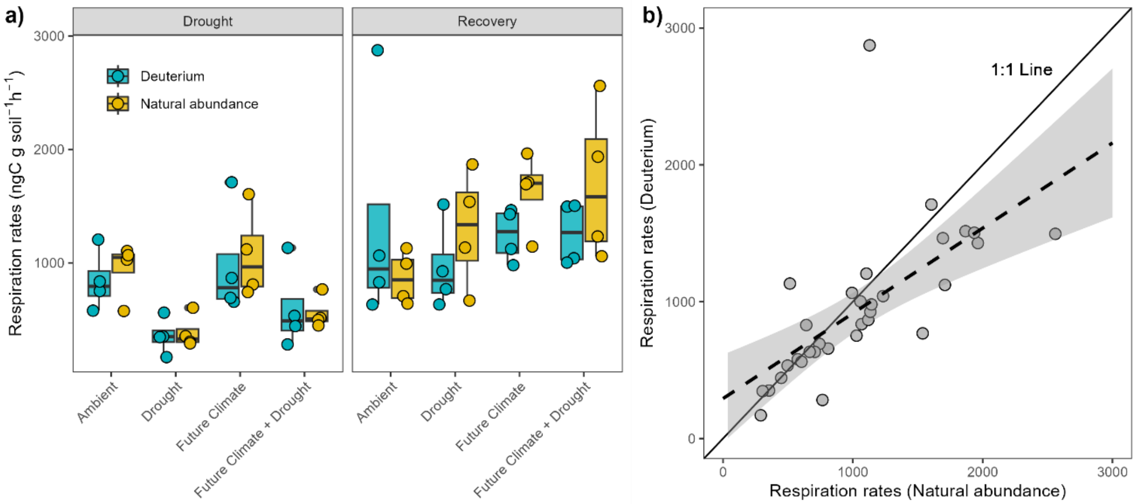

Supplementary Figure 13. Comparison of respiration rates determined in samples incubated with natural abundance vs. ^{2}H labelled water. a) Total respiration rate (in ng C g soil^{-1} h^{-1}) obtained from soil samples incubated with natural abundance water (yellow) and ^{2}H-labelled water (blue) for all different treatments and time periods. Statistical analysis (two sided paired t-test) revealed no significant differences. Box center line represents the median, the box limits the upper and lower quartiles, whiskers the 1.5x interquartile range; separated points represent potential outliers. The sample size 'n' represents biologically independent samples (n=4 replicates). b) Correlation of respiration rates of samples incubated with natural abundance and ^{2}H-labelled water (both given in ng C g soil^{-1} h^{-1}). The solid line represents the 1:1 line, the dashed line represent the best fit line.

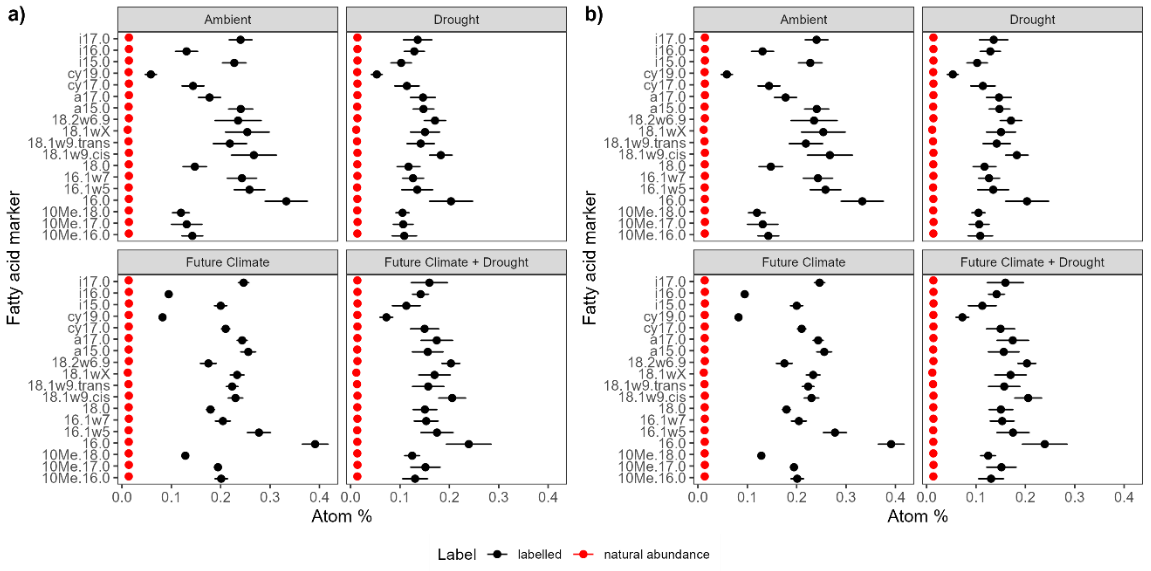

Supplementary Figure 14: Compound-specific isotope composition (natural abundance in red) and enrichment (labelled in black) of the FAMEs included in our study reported in ^{2}H atom% for a) the 'Drought' and b) the 'Recovery' period. For each period, the data is grouped by treatment (ambient, drought, future climate and future climate + drought). Values are shown as average ^{2}H atom % (error bars show standard errors, the sample size represents biologically independent samples; n=4 replicates).

Supplementary Table 1. Impacts of drought and future climate on soil water content in soils during Drought and Recovery. Average soil water content was measured gravimetrically (± standard error, n=4 replicates).

	Soil water content (g H_2O g^{-1} dry soil)			
	'Drought'		'Recovery'	
Factor	Average	Standard error	Average	Standard error
Ambient	0.32	0.010	0.33	0.008
Drought	0.08	0.010	0.30	0.013
Future Climate	0.31	0.008	0.32	0.005
Future Climate + Drought	0.06	0.009	0.30	0.007

Supplementary Table 2: Effect of drought and future climate and their interactions on mass-specific growth, mass-specific respiration (both in mg C g microbial C^{-1} h^{-1}) and CUE determined by ^{2}H- and ^{18}O-vapor SIP, respectively. We used linear mixed models (as described in the Method section) with drought and future climate as fixed factor, and plot number as random factor to account for plot variability to test impacts of the treatments during Drought and Recovery, separately; p-values < 0.05 are given in bold (n = 4 replicates per treatment).

	Growth							
	'Drought'				'Recovery'			
	^{2}H-PLFA		^{18}O-DNA		^{2}H-PLFA		^{18}O-DNA	
Factor	F	p	F	p	F	p	F	p
Drought	15.061	**0.002**	48.878	**<.0001**	0.538	0.477	0.088	0.771
Future Climate	0.085	0.775	0.046	0.832	0.179	0.679	3.988	0.069
Interaction	0.004	0.945	0.117	0.737	0.319	0.582	0.0002	0.988
	Respiration							
	'Drought'				'Recovery'			
	^{2}H-PLFA		^{18}O-DNA		^{2}H-PLFA		^{18}O-DNA	
Drought	7.727	**0.016**	12.436	**0.004**	0.019	0.892	1.324	0.272
Future Climate	3.770	0.076	1.204	0.293	20.634	**0.0007**	27.394	**0.0002**
Interaction	0.531	0.479	0.001	0.970	1.508	0.242	0.079	0.782
	CUE							
	'Drought'				'Recovery'			
	^{2}H-PLFA		^{18}O-DNA		^{2}H-PLFA		^{18}O-DNA	
Factor	F	p	F	p	F	p	F	p
Drought	0.003	0.951	0.256	0.621	0.049	0.826	3.027	0.107
Future Climate	3.610	0.081	3.395	0.090	99.734	**<0.0001**	32.931	**0.0001**
Interaction	2.287	0.156	1.034	0.329	1.778	0.106	0.734	0.408

Supplementary Table 3. Effects of drought and future climate and their interaction on PLFA community composition (relative abundance) and relative PLFA-based growth (growth rates), using a permutational ANOVA (permanova) based on Euclidean distance matrices as described in the Results section, separately done for the Drought and Recovery period. p-values < 0.05 are given in bold (n = 4 replicates per treatment; Df = degrees of freedom).

	‘Drought’					
	Abundance			Growth rates		
	Df	R^2	*p*	Df	R^2	*p*
Drought	1	0.297	**0.001**	1	0.405	**0.001**
Future Climate	1	0.139	**0.019**	1	0.246	**0.001**
Interaction	1	0.047	0.341	1	0.065	**0.040**
	‘Recovery’					
	Abundance			Growth rates		
	Df	R^2	*p*	Df	R^2	*p*
Drought	1	0.181	**0.002**	1	0.140	**0.034**
Future Climate	1	0.119	**0.012**	1	0.070	0.330
Interaction	1	0.094	0.064	1	0.077	0.281

Supplementary Table 4. Effects of the drought and future climate treatment and their interactions during the Drought and Recovery period on different microbial groups' mass-specific growth rates by ^2H incorporation into PLFA (in mg C g microbial C^{-1} h^{-1}) were tested using linear mixed effect models (as described in the Methods section), separately for fungi, gram negative and gram positive bacteria, actinobacteria, F:B ratio (fungi to bacteria marker ratio) and for general biomarkers (General). Drought and future climate were used as fixed factors, and plot number as random factor to account for plot variability to test impacts of the treatments separately during 'Drought' and 'Recovery'. Effects were considered significant when p values < 0.05 and are given in bold (n = 4 replicates per treatment).

	'Drought'					
Factor	Drought		Future Climate		Interaction	
	F	p	F	p	F	p
Fungi	0.176	0.683	1.426	0.256	0.327	0.578
Gram negative	23.830	**<0.0001**	0.414	0.532	0.015	0.905
Gram positive	20.468	**0.0007**	0.486	0.498	0.023	0.881
F:B ratio	31.299	**<0.0001**	0.712	0.415	0.961	0.346
Actinobacteria	7.232	**0.019**	2.756	0.122	1.220	0.290
General	29.215	**0.0002**	0.038	0.846	0.000	0.999

	'Recovery'					
Factor	Drought		Future Climate		Interaction	
	F	p	F	p	F	p
Fungi	1.075	0.320	0.319	0.583	1.615	0.228
Gram negative	0.194	0.668	0.038	0.849	0.181	0.678
Gram positive	0.052	0.823	0.027	0.871	0.174	0.683
F:B ratio	0.597	0.454	0.589	0.457	0.929	0.345
Actinobacteria	0.034	0.856	0.006	0.936	0.207	0.656
General	0.390	0.543	0.270	0.612	0.171	0.686

Supplementary Table 5. Impacts of drought and future climate and their interaction on absolute (given in ng C g soil^{-1} h^{-1}) and relative (% of PLFA) NLFA production during the Drought and Recovery period. Effects were tested using linear mixed effect models (as described in detail in the Method section), with drought and future climate as fixed factors, and plot number as random factor to account for plot variability, for fungal, gram negative bacterial and total (sum of all) biomarkers. p values < 0.05 are given in bold (n = 4 replicates per treatment).

	'Drought'							
	Fungi (ng C g C^{-1} h^{-1})		Gram negative (ng C g C^{-1} h^{-1})		Fungi (% of PLFA)		Gram negative (% of PLFA)	
Factor	F	p	F	p	F	p	F	p
Drought	22.485	**0.0005**	16.717	**0.015**	87.339	**<.0001**	66.491	**<.0001**
Future Climate	0.373	0.552	0.004	0.948	0.527	0.481	2.249	0.159
Interaction	0.147	0.707	0.121	0.733	0.200	0.662	0.511	0.488
	'Recovery'							
	Fungi (ng C g C^{-1} h^{-1})		Gram negative (ng C g C^{-1} h^{-1})		Fungi (% of PLFA)		Gram negative (% of PLFA)	
Factor	F	p	F	p	F	p	F	p
Drought	0.014	0.905	0.580	0.460	0.607	0.450	0.635	0.442
Future Climate	0.408	0.534	0.0001	0.989	1.215	0.291	0.396	0.540
Interaction	0.005	0.940	0.760	0.400	0.300	0.593	1.088	0.317

Supplementary Table 6. Impacts of drought and future climate and their interaction on NLFA amount (nmol C g soil^{-1}) during the Drought and Recovery period. Effects were tested using linear mixed effect models (as described in detail in the Method section), with drought and future climate as fixed factors, and plot number as random factor to account for plot variability, for fungal and total (sum of all) biomarkers. p values < 0.05 are given in bold (n = 4 replicates per treatment).

	'Drought'			
	Fungi (nmol C g C^{-1})		Total (nmol C g C^{-1})	
Factor	F	P	F	p
Drought	16.337	**0.002**	0.996	0.337
Future Climate	4.602	0.053	0.384	0.546
Interaction	5.553	**0.036**	11.401	**0.005**
	'Recovery'			
	Fungi (nmol C g C^{-1})		Total (nmol C g C^{-1})	
Factor	F	p	F	p
Drought	0.028	0.869	1.466	0.249
Future Climate	0.089	0.770	0.006	0.936
Interaction	1.179	0.298	2.590	0.133
