# Supplementary material for: Soil fungi remain active and invest in storage compounds during drought independent of future climate conditions
Source: Nat Commun. 2024 Nov 29;15:10410. doi: 10.1038/s41467-024-54537-y (PMC11607446; doi:10.1038/s41467-024-54537-y)
Supplement: Supplementary file 3 — Description of Additional Supplementary Files [file 41467_2024_54537_MOESM3_ESM.pdf]

Description of Additional Supplementary Files

File Name: Supplementary Data 1
Description: Isotopic enrichment for each samples from PLFA, NLFA and DNA analyses
