# Supplementary material for: Soil fungi remain active and invest in storage compounds during drought independent of future climate conditions
Source: Nat Commun. 2024 Nov 29;15:10410. doi: 10.1038/s41467-024-54537-y (PMC11607446; doi:10.1038/s41467-024-54537-y)
Supplement: Supplementary file 5 — Reporting Summary [file 41467_2024_54537_MOESM5_ESM.pdf]

nature portfolio

Corresponding author(s): Alberto Canarini, Lucia Fuchslueger, Andreas Richter

Last updated by author(s): Nov 6, 2024

Reporting Summary

Nature Portfolio wishes to improve the reproducibility of the work that we publish. This form provides structure for consistency and transparency in reporting. For further information on Nature Portfolio policies, see our Editorial Policies and the Editorial Policy Checklist.

Statistics

For all statistical analyses, confirm that the following items are present in the figure legend, table legend, main text, or Methods section.

n/a	Confirmed	
☐	☒	The exact sample size (*n*) for each experimental group/condition, given as a discrete number and unit of measurement
☐	☒	A statement on whether measurements were taken from distinct samples or whether the same sample was measured repeatedly
☐	☒	The statistical test(s) used AND whether they are one- or two-sided<br>*Only common tests should be described solely by name; describe more complex techniques in the Methods section.*
☒	☐	A description of all covariates tested
☐	☒	A description of any assumptions or corrections, such as tests of normality and adjustment for multiple comparisons
☐	☒	A full description of the statistical parameters including central tendency (e.g. means) or other basic estimates (e.g. regression coefficient) AND variation (e.g. standard deviation) or associated estimates of uncertainty (e.g. confidence intervals)
☐	☒	For null hypothesis testing, the test statistic (e.g. *F*, *t*, *r*) with confidence intervals, effect sizes, degrees of freedom and *P* value noted<br>*Give P values as exact values whenever suitable.*
☒	☐	For Bayesian analysis, information on the choice of priors and Markov chain Monte Carlo settings
☒	☐	For hierarchical and complex designs, identification of the appropriate level for tests and full reporting of outcomes
☐	☒	Estimates of effect sizes (e.g. Cohen's *d*, Pearson's *r*), indicating how they were calculated

Our web collection on statistics for biologists contains articles on many of the points above.

Software and code

Policy information about availability of computer code

Data collection	No software was used to collect the data
Data analysis	The R software (version 3.6.3) was used for statistical analyses and figures. We used 'ggplot2' for graphs. We used the 'lme' function (from the package nlme) to test the differences in treatments. Differences in respiration between samples where 2H was added compared to natural abundance water were evaluated via two-tailed paired t-test, via the R function 't.test'. Correlations between CUE and mass specific growth were assessed via Pearson's correlation via the 'stat_cor' function. Principal component analyses o were assessed via the function 'PCA' of the package 'FactoMineR'. Permanova analysis was carried with the funciton 'adonis'.<br>We described the usage of each software in details in the Methods section.

For manuscripts utilizing custom algorithms or software that are central to the research but not yet described in published literature, software must be made available to editors and reviewers. We strongly encourage code deposition in a community repository (e.g. GitHub). See the Nature Portfolio guidelines for submitting code & software for further information.

Data

Policy information about availability of data

All manuscripts must include a data availability statement. This statement should provide the following information, where applicable:

- Accession codes, unique identifiers, or web links for publicly available datasets
- A description of any restrictions on data availability
- For clinical datasets or third party data, please ensure that the statement adheres to our policy

The data generated in this study are provided in the Supplementary Information. The code is made public via Github repository under the DOI: https://doi.org/10.5281/zenodo.14048057

Research involving human participants, their data, or biological material

Policy information about studies with human participants or human data. See also policy information about sex, gender (identity/presentation), and sexual orientation and race, ethnicity and racism.

Reporting on sex and gender	*Use the terms sex (biological attribute) and gender (shaped by social and cultural circumstances) carefully in order to avoid confusing both terms. Indicate if findings apply to only one sex or gender; describe whether sex and gender were considered in study design; whether sex and/or gender was determined based on self-reporting or assigned and methods used.*<br>*Provide in the source data disaggregated sex and gender data, where this information has been collected, and if consent has been obtained for sharing of individual-level data; provide overall numbers in this Reporting Summary. Please state if this information has not been collected.*<br>*Report sex- and gender-based analyses where performed, justify reasons for lack of sex- and gender-based analysis.*
Reporting on race, ethnicity, or other socially relevant groupings	*Please specify the socially constructed or socially relevant categorization variable(s) used in your manuscript and explain why they were used. Please note that such variables should not be used as proxies for other socially constructed/relevant variables (for example, race or ethnicity should not be used as a proxy for socioeconomic status).*<br>*Provide clear definitions of the relevant terms used, how they were provided (by the participants/respondents, the researchers, or third parties), and the method(s) used to classify people into the different categories (e.g. self-report, census or administrative data, social media data, etc.)*<br>*Please provide details about how you controlled for confounding variables in your analyses.*
Population characteristics	*Describe the covariate-relevant population characteristics of the human research participants (e.g. age, genotypic information, past and current diagnosis and treatment categories). If you filled out the behavioural & social sciences study design questions and have nothing to add here, write "See above."*
Recruitment	*Describe how participants were recruited. Outline any potential self-selection bias or other biases that may be present and how these are likely to impact results.*
Ethics oversight	*Identify the organization(s) that approved the study protocol.*

Note that full information on the approval of the study protocol must also be provided in the manuscript.

Field-specific reporting

Please select the one below that is the best fit for your research. If you are not sure, read the appropriate sections before making your selection.

☐ Life sciences ☐ Behavioural & social sciences ☒ Ecological, evolutionary & environmental sciences

For a reference copy of the document with all sections, see nature.com/documents/nr-reporting-summary-flat.pdf

Ecological, evolutionary & environmental sciences study design

All studies must disclose on these points even when the disclosure is negative.

Study description	This field experiment (ClimGrass) explored the effects of climate change (increased atmospheric CO2, temperatures and drought events) on soil microbial community, physiological parameters (growth, respiration and carbon use efficiency). The ClimGrass project entails 54 plots with a combined warming and Free-Air-Carbon dioxide-Enrichment (T-FACE) setup, put into full operation in 2014 to manipulate temperature and CO2 at three levels each. Fully automated rainout shelters were installed above half of the ambient and above half of the combined +3.0 °C and +300 ppm CO2 (i.e.,'Future Climate') plots. All plots are harvested (plant biomass) three times a year (spring, summer and autumn) and receive identical rates of mineral fertilizer, applied in three batches giving a total load of 90 kg N, 65 kg P and 170 kg K per hectare and year.<br>For this experiment, we selected 16 plots representing four different treatments in a full factorial design (n=4 per treatment, respectively): ambient ('ambient'), drought ('drought'), eCO2 and elevated temperature combined (+300ppm +3°C; 'future climate'), and future climate with drought ('future climate + drought'). The drought period was simulated in the field between June 17th 2020 until August 3rd 2020 by excluding all naturally occurring precipitation. The drought plots then received a scheduled rewetting with 40 mm of previously collected rainwater on August 3rd 2020, after which the automatic rain-out shelters were switched off and the

plots were used to investigate the recovery from drought.
Subsamples of soil were incubated (48 hours) with 2H or 18O labelled water and further extracted to assess physiological metrics (growth, respiration and carbon use efficiency). The remaining soil was immediately used to measure water content and for extractions of available nutrients, carbon and microbial biomass.

Research sample	Topsoil samples were collected from grassland plots at each time point. Three soil cores (10 cm deep, 2 cm diameter) were collected at each plot and passed through a 2 mm sieve. The topsoil was chosen since it is the most active soil horizon that is responsible for the majority of microbial activity and associated biogeochemical cycling, and will be most affected by drought.
Sampling strategy	The site used in this study is a permanent long-term experiment investigating effects of climate change on a mountain grassland ecosystem, established in 2013 and fully functional since 2014. Four replicates per treatment (and control) were chosen. The design strategy takes account of logistic constraints imposing limitations on the number of plots with a rain out shelter, minimizing the number of replicate necessary to allow statistical evaluation of the hypotheses tested. At each plot three soil cores were collected (spaced at roughly 30 cm from each other). Samples were taken at two different time points: towards the end of the drought period (29th of July) and two days after the rewetting event (5th of August), to follow drought and rewetting dynamics.
Data collection	Data was recorded automatically by the machines used and downloaded as an excel sheet (or transformed into excel sheet format before analysis in the R software).
Timing and spatial scale	The grassland at the field site represents a typical mountain grassland of many parts of the Alps, in regards to both climatic conditions and management (MAT:8.5 ^{o}C and MAP: 1077 mm). Plots were arranged in a randomized design. Plots were at least 2 m apart. In each plot (n=4 for each treatment) three replicate soil cores (2 cm diameter) were collected to a depth of 0-10 cm and were mixed. Samples transported to the University of Vienna and sieved, and incubated at their respective field temperatures measured at the time of harvest. Laboratory analysis were carried out within two days following arrival at the University of Vienna.
Data exclusions	No data was excluded from the analysis.
Reproducibility	Standard approaches, materials, machines, and methodology were used, permitting future reproduction.
Randomization	The experimental design is based on a completely randomized design. Portion of the field where randomized into experimental groups by initially dividing the field into plots and then blindly choosing plots to allocate to the experimental groups.
Blinding	Data was collected blindly as to each sample was assigned a number without any reference to treatment. Data analysis was not blinded as treatment information were necessary to apply specific analysis (e.g. linear mixed effect model).

Did the study involve field work? ☒ Yes ☐ No

Field work, collection and transport

Field conditions	Samples were collected from a managed montane grassland as part of a multifactorial climate change experiment ('ClimGrass') located at the Agricultural Research and Education Center (AREC) in Raumberg-Gumpenstein. The site is characterized by a mean annual temperature of 8.5 ^{o}C and a mean annual precipitation of 1077 mm. According to the WRB-system (81) the soil is classified as Dystric Cambisol (arenic, humic) with a loamy sand texture and a pH-value of ~5.5. Before establishment of the 'ClimGrass' experiment, a typical grassland mixture was sown in an area of homogeneous soils in 2007 (the species list is provided in the supplementary text). The ClimGrass project entails 54 plots with a combined warming and Free-Air-Carbon dioxide-Enrichment (T-FACE) setup, put into full operation in 2014 to manipulate temperature and CO2 at three levels each. Fully automated rainout shelters were installed above half of the ambient and above half of the combined +3.0 °C and +300 ppm CO2 plots. All plots are harvested (plant biomass) three times a year (spring, summer and autumn) and receive identical rates of mineral fertilizer, applied in three batches giving a total load of 90 kg N, 65 kg P and 170 kg K per hectare and year.
Location	The study site is located in the Austrian Alps, Styria, Austria (47°29'38''N, 14°06'03''E).
Access & import/export	All plots were accessible by car/walking and we were accompanied by local scientists or employees working for the Agricultural Research and Education Center (AREC), which manages the site. Soil samples were all collected within Austria and no permits were necessary for transportation.
Disturbance	When the experiment was constructed, disturbance was not avoidable. To reduce disturbance on the plots at the time of sampling only one person was allowed to collect soil samples.

Reporting for specific materials, systems and methods

We require information from authors about some types of materials, experimental systems and methods used in many studies. Here, indicate whether each material, system or method listed is relevant to your study. If you are not sure if a list item applies to your research, read the appropriate section before selecting a response.

Materials & experimental systems

n/a	Involved in the study
☒	☐ Antibodies
☒	☐ Eukaryotic cell lines
☒	☐ Palaeontology and archaeology
☐	☒ Animals and other organisms
☒	☐ Clinical data
☒	☐ Dual use research of concern
☒	☐ Plants

Methods

n/a	Involved in the study
☒	☐ ChIP-seq
☒	☐ Flow cytometry
☒	☐ MRI-based neuroimaging

Animals and other research organisms

Policy information about studies involving animals; ARRIVE guidelines recommended for reporting animal research, and Sex and Gender in Research

Laboratory animals	This study did not involve laboratory animals.
Wild animals	Wild animals were not used in this study.
Reporting on sex	This study did not involve animals.
Field-collected samples	Field-collected soil samples were immediately transported to the University of Vienna and stored in airtight plastic bags for transportation. Samples were stored for about 2 days in incubators at the respective field temperature at the time of harvest. After completion of all analyses, soil samples were air-dried and stored at room temperature in the dark.
Ethics oversight	No ethical approval or guidance was required. We did not work with dangerous nor foreign materials (i.e. exotic species, pathogens, etc.).

Note that full information on the approval of the study protocol must also be provided in the manuscript.

Plants

Seed stocks	This study did not involve plants
Novel plant genotypes	This study did not involve plants
Authentication	This study did not involve plants

April 2023
